# Ethnobotanical Study of Medicinal Plants Used as Therapeutic Agents to Manage Diseases of Humans

**DOI:** 10.1155/2022/4104772

**Published:** 2022-02-17

**Authors:** Sanae Achour, Mohamed Chebaibi, Hanane Essabouni, Mohammed Bourhia, Lahcen Ouahmane, Ahmad Mohammad Salamatullah, Mourad A M Aboul-Soud, John P. Giesy

**Affiliations:** ^1^Laboratory of Pharmacology and Toxicology, University Hospital Hassan II, Fez, Morocco; ^2^Biomedical and Translational Research Laboratory, Faculty of Medicine and Pharmacy of the Fez, University of Sidi Mohamed Ben Abdellah, BP 1893, Km 22, Road of Sidi Harazem, Fez, Morocco; ^3^Department of Biology, Faculty of Sciences Dhar El Meharz, University of Sidi Mohamed Ben Abdellah, Fez, Morocco; ^4^Laboratory of Microbial Biotechnology, Agro-Sciences and Environment (BioMAgE), Cadi Ayyad University, Marrakesh, Morocco; ^5^Department of Food Science & Nutrition, College of Food and Agricultural Sciences, King Saud University, P. O. Box 2460, Riyadh 11451, Saudi Arabia; ^6^Department of Clinical Laboratory Sciences, College of Applied Medical Sciences, King Saud University, P.O. Box 10219, Riyadh 11433, Saudi Arabia; ^7^Toxicology Centre, University of Saskatchewan, 44 Campus Drive, Saskatoon, SK, Canada S7N 5B3; ^8^Department of Veterinary Biomedical Sciences, University of Saskatchewan, Saskatoon, SK, Canada S7N 5B4; ^9^Department of Integrative Biology, Center for Integrative Toxicology, Michigan State University, East Lansing, MI 48824, USA; ^10^Department of Environmental Science, Baylor University, One Bear Place #97266, Waco, TX 76798-7266, USA

## Abstract

**Objective:**

This work aimed to survey medicinal plants used in phytotherapy in the Fez-Boulemane region, Morocco.

**Methods:**

A comprehensive ethnobotanical survey was conducted using a questionnaire to collect data from thirty herbalists on medicinal plants used for therapeutic purposes in the Fez-Boulemane region, Morocco.

**Results:**

The mean age of herbalists interviewed was 52.13 years. Forty percent of the herbalists were illiterate, and 73% referred to the experiences of their parents as knowledge of the properties and uses of medicinal plants. One hundred and eight medicinal plants belonging to 51 botanical families were recommended by herbalists in the region of Fez-Boulemane, Morocco, for treatment. According to the respondents, *Lawsonia inermis* L., *Rosmarinus officinalis* L., and *Lavandula coronopifolia* L. were the most used plants with the percentage of 13%, 12%, and 11%, respectively. Most plants had been involved in the treatment of digestive disorders (25%) and osteoarticular diseases (24%). Some (7.4%) of the plants mentioned in our survey were potentially toxic.

**Conclusion:**

It was learned that irrational use of toxic plants and unknown compositions of recipes are recommended to consumers. As a result, particular attention should be paid to risks related to plants used in traditional treatment without scientific validation. It is envisaged that increasing awareness, by conducting educational campaigns and transferring evidence-based scientific knowledge, on traditional treatments among the local population is expected to have beneficial impacts on health and disease management.

## 1. Introduction

Traditional medicine is very ancient, and it is the sum of all knowledge, skills, and practices based on the theories, beliefs, and experiences specific to various cultures, whether explicable or not, and for which is used in the preservation of health, as well as in the prevention, diagnosis, improvement, or treatment of physical and mental illnesses [1]. The therapeutic effects of medicinal plants and their uses in several medicines encourage people to use herbal medicine to cure physical and mental illnesses. Morocco, by its geographical position and climate conditions, has a rich and varied flora, made up of more than 4,200 species, including 500 to 600 species that are used in traditional medicine [2, 3].

Medicinal plants are widely used by the Moroccan population to cure diseases [4–6]. Illiteracy, the limited income of the Moroccan population, and sociocultural factors, in general, have resulted in a relatively large demand for treatment using plants. This frequent use is due to the belief of people that plants are natural products and have no adverse or toxic effects [7, 8]. Products used are often a “variegation” of plants; the knowledge and requirements of preparation and consumption are generally not mastered [9]. Previous studies conducted by the Anti-Poison and Pharmacovigilance Center in Morocco (CAPM) have shown that plants are involved in 3 to 5% of all poisoning across the country [9, 10]. There is no official, traditional, and well-coded pharmacopeia for Morocco. Due to the absence of legislation and control, limitations such as little regulation of collection, sale, or use of plants exist. The lack of any formal studies of the medicinal plants used in Morocco has also limited the valuation of these species and development of their use by modern medicine [9]. There was no systematic listing of plants used in medicine in Morocco, and much of the information available was or an oral nature with little committed to writing. For the above reasons, it was deemed necessary to conduct an ethnobotanical survey among herbalists of the Fez-Boulemane region of Morocco in order to collect as much information as possible concerning the therapeutic uses of medicinal plants used by the indigenous population of the Fez-Boulemane-Morocco region.

## 2. Materials and Methods

### 2.1. Study Area

**Figure 1:**
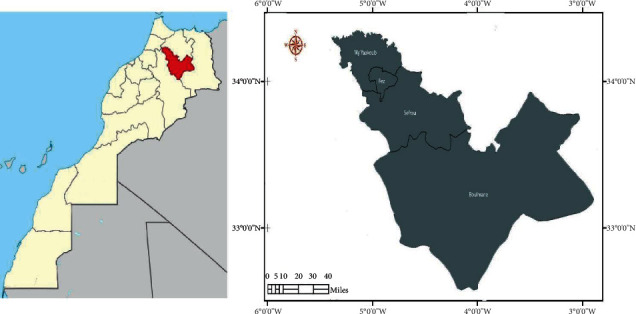
Map of the study area, prepared by QGIS software.

The region of Fez-Boulemane is a part of the Middle Atlas, Morocco (Figure 1), which is located in the center-north of the Kingdom. It is considered as a crossroad between the east and northeast of the Kingdom. The Fez-Boulemane region is subject to three climates: a continental climate in the north, a cold and humid climate in mountainous areas, and a semiarid climate in the high hills of Boulemane. The region also includes one of the largest forests in Morocco and extends over most of the northern slope of the elevated and mountainous plateau, extending from Imilchil to Midelt, known as the Eastern High Atlas. Thus, due to its geographical position and its climatic conditions, this region has a rich and varied flora that includes several species used in traditional medicine.

### 2.2. Data Collection

Between April and September 2016, an ethnobotanical survey was conducted for four provinces in the Fez-Boulemane region, including Moulay Yacoub, Fez, Sefrou, and Boulemane. The questionnaire was divided into two parts; the first concerned sociodemographic and professional parameters of herbalists, including age, sex, locality, level of education, years of experience, and source of acquisition of knowledge; the second part was reserved for ethnobotanical indices, including plants used, methods of preparation, and pathologies treated.

### 2.3. Identification of Plants

Identification of botanical names has been verified following the “Flore Practique du Maroc” (Practical Flora of Morocco). Each plant has been registered under a specific number and deposited at the Herbarium of Biomedical and Translational Research Laboratory (BTRL), Sidi Mohammed Ben Abdellah University, Fez, Morocco.

### 2.4. Statistical Analyses

The sociodemographic data were analyzed by a simple descriptive statistical analysis using percentages and frequencies. Ethnobotanical data were analyzed using percentages and relative frequency of citation. Data entry and statistical analysis were performed by SPSS v.21 software.

## 3. Results

### 3.1. Sociodemographic and Professional Parameters

#### 3.1.1. Sociodemographic Data

Among the thirty herbalists interviewed, the age varied between 20 years and 70 years, with an average age of 52.13 ± 13.17 years. Eighty-seven percent of herbalists questioned were in urban areas, with the other 13% in rural areas. Forty percent of herbalists were illiterate, and 20% had a primary school and 10% had a secondary school education (Table 1).

### 3.2. Professional Data

Fifteen herbalists (50%) had professional experience between 13 and 20 years, and 7 of them had been practicing this profession for more than 20 years. Twenty-eight of the herbalists had received no training in herbal medicine. Concerning the source of information, 73% of herbalists refer to the experiences of their parents to use medicinal plants as remedies for specific diseases, 18% use radio broadcasts, and 7% use books. This study showed that 23 of the herbalists (77%) are not members of any association of herbalists, while 7 (23%) are registered in associations (National Authority of Herbalists in Morocco) (Table 2).

### 3.3. Ethnobotanical Data

#### 3.3.1. Collection and Conservation of Plants

Twenty-six of the herbalists (87%) bought plants from distributors, and 13% of them reported that they picked some plants from forests according to a calendar open all year. Most herbalists (22%) kept plants in a dry place, 19% in glass bottles, 13% in cloth bags, and 5% in plastic bags, and only 8% is kept away from light. Concerning the duration of the conservation of plants, it never exceeds 1 year.

#### 3.3.2. Mode of Utilization of Medicinal Plants

**Figure 2:**
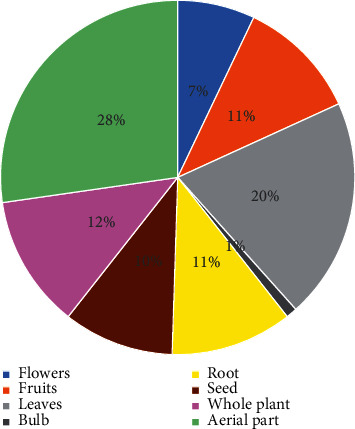
Parts of plants used by herbalists inFez-Boulemane region, Morocco.

**Figure 3:**
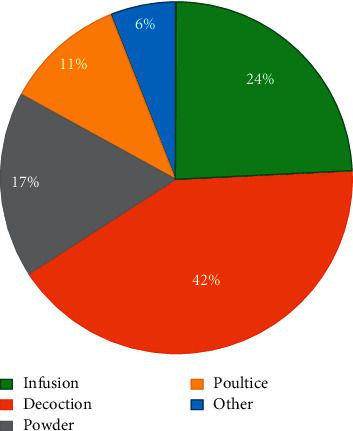
Methods of preparation cited by herbalists in the region of Fez-Boulemane.

Method of preparation, parts used, and route of administration: in total, nine parts of plants are used in traditional medicine including the seed, roots, whole plant, leaves, flower, aerial part, gum, and fruit. The percentage of use of these different parts showed that the leaves and the aerial part are the most cited with 20% and 27%, respectively. The whole plant, fruit, and root take the second place with a respective percentage of 12% and 11% (Figure 2). Decoction and infusion are the two most used preparation methods with 42% and 24%, respectively (Figure 3). The oral route was the most used (60%), followed by the cutaneous route (31%). Most customers (67%) request prepared mixtures, and 33% prefer the use of separate medicinal plants.

Among the most demanded plants by customers, we note *Lawsonia inermis* L. (13%) and *Rosmarinus officinalis* L. (12%), followed by *Carum carvi* L. and *Lavandula coronopifolia* L. (11%) (Table 3).

Pathologies treated with plants: most people use medicinal plants for the treatment of digestive disorders (25%), osteoarticular diseases (24%), and urogenital diseases (12%) (Table 4). Several groups of medicinal plants are used in the treatment of diseases in the region of Fez-Boulemane (Table 5).

#### 3.3.3. Toxic Plants and Mixtures

Toxic plants recommended by herbalists: some plants used by the local population for treatments can cause toxic effects. These included *Atractylis gummifera* L. (15%) followed by *Peganum harmala* L. (14%) and *Papaver somniferum* L. (12%) (Table 6).

Mixtures proposed by herbalists: it was difficult to obtain information on the mixtures because the herbalists' answers remained vague and evasive; herbalists prefer to sell ground and prepared mixtures. For fairly well-known mixtures, herbalists sell recipes without description, and the components are always sold in a powder format (Table 7).

## 4. Discussion

According to floristic and ethnobotanical studies of the medicinal flora of the Eastern High Atlas conducted between 2012 and 2013, only 20% of the population preferred modern medicine [11]. In southeastern Morocco, 70.7% of individuals practice phytotherapy [12]. The results of the two studies confirm ours since 75.1% of the population studied use phytotherapy. A total of 108 species belonging to 51 families, of which Lamiaceae, Apiaceae, and Asteraceae are the most represented medicinal plants used by the population of the region. These results were consistent with those of a survey conducted in eastern Morocco [13], which showed that the most represented botanical families in eastern Morocco are 53 species of Asteraceae, 34 species of Lamiaceae, 29 species of Fabaceae, 28 species of Apiaceae, 17 species of Liliaceae, and 17 species of Poaceae. Among the 108 species used in phytotherapy, the most often identified are the same species used by the population of the Rabat region of Morocco [14].

While various parts of plants are used, aerial parts were the most used part, with 27.5%, followed by the leaves with 20.0%. According to several floristic and ethnobotanical studies of medicinal plants, aerial parts are the parts most used in phytotherapy [11, 15]. The use of aerial parts, including stems and leaves or leaves only, can be explained by ease of harvesting and also availability of these parts throughout the year. Another reason is that the aerial part is the seat of photosynthesis [16–18].

Various therapeutic practices are used by local populations, namely, decoction, infusion, powdered preparation, fumigation, poultice, maceration, raw, and cooking. Most plants are involved in the treatment of digestive disorders (18.3%), pathologies linked to metabolism and secretion (14.7%), pathologies of the respiratory system (11.4%), and also bone and joint diseases (10%). Our results are in agreement with the works of Tahri et al. [15]. The methods for preparation mostly are decoction, infusion, and powder, with a rate of 39.93%, 26.46%, and 16.63%, respectively. It has been reported that preparations of plants are administered internally, via oral ingestion or rectally by enemas, in 65.71% of cases, or externally, by local application, in 31.42% [19]. This agrees with our study where the oral route remains the main mode of use of plants (66.4%) of the time. More than 50% of the Moroccan population uses combinations of two or more plants, which are, in most cases, sold by herbalists.

Medicinal plants are complex mixtures of various molecules. Their composition is often unknown and made up of molecules with known biological activity, including heterosides, alkaloids, anthocyanins, tannins, and steroids. These constituents can, at sufficient concentrations, cause toxicity via multiple mechanisms of action [20]. Combining plants can be used as a mechanism for masking the toxicity of herbal preparations in several African countries [21]. Contents of these constituents can “naturally” vary from one preparation to another; among the plants used, 14.8% are toxic. The study was conducted on uses of medicinal plants in the circle of Mechra Bel Ksiri region of western Morocco, which showed that only 27% of the population, especially the oldest subjects, understand that plants can be toxic [13]. Alternatively, in the study presented here, 96.7% of people using medicinal plants in the Fez-Boulemane region, Morocco, understood the concept of toxicity and that some plants could be toxic. The best-known toxic plant is thistle, which is no longer used for therapeutic purposes. Three hundred and twenty-eight or 54.6% of users of medicinal plants have observed undesirable effects, including toxicity or aggravation of the disease. This can be explained by the lack of awareness among tutors about risks of toxic effects associated with the use of plants or by uninformed use of plants, without respecting the dose, the parts used, nor the method of preparation. Toxicities of several plants used by the population of the region of Fez-Boulemane of Morocco including *Atractylis gummifera* L. have been demonstrated in controlled clinical studies. Toxicity of this plant is due to two diterpene glucosides, atractyloside and carboxyatractyloside, which inhibit mitochondrial oxidative phosphorylation [22]. *Herniaria hirsuta* is another nephrotoxic plant that is the cause of several cases of renal lithiasis received by the Hassan II hospital center in Fez (CHU Fez) [23]. Use of *Aristolochia longa* L. as an anticancer plant in Morocco can, due to aristolochic acid, cause interstitial nephritis and DNA adducts to the kidney [23]. Interstitial nephritis is also referred to as tubulointerstitial nephritis and is inflammation of the area of the kidney known as the renal interstitium, which consists of a collection of cells, extracellular matrix, and fluid surrounding the renal tubules. Most of the harmful effects of medicinal plants are not due to inherent toxicities of medicinal plants, but rather are due to errors in identification, involuntary contamination, and noncompliance with the adequate dose or interactions with drugs [19]. In this survey, 61.7% of the population uses plants in combination with synthetic pharmaceuticals, which can increase risks of side effects and can result in frank toxicities. Pharmacokinetic interactions of herbal products can affect absorption, distribution, and elimination of certain prescribed drugs. In other cases, interactions can promote increases in concentrations and cause undesirable side effects.

## 5. Conclusions

Morocco has a rich and variable flora, which, in part, explains the frequent use of plants for therapeutic, cosmetic, and gastronomic purposes. However, the irrational, anarchic, and uncontrolled consumption of plants can be responsible for poisoning, which can be life-threatening. Thus, our study allowed us to describe the relative importance given to the use of phytotherapy by the population of the region of Fez-Boulemane and to confirm the persistent use of plants for therapeutic purposes, despite the revolution of medical technology and availabilities of modern synthetic pharmaceuticals. It was learned that toxic plants and unknown compositions of recipes are also sold to consumers. Hence, the interest in raising awareness among population about the dangers is associated with the use of nonscientifically validated plants for treatment.

## Figures and Tables

**Table 1:** Distribution of herbalists according to sociodemographic characteristics.

Variables	Number of respondents Proportion (%)	
Sex		
Male	28	93
Female	2	7
Locality		
City	26	87
Village	4	13
Age (years)		
<25	1	3
25–45	5	17
>45	24	80
Mean age	52.13	
Level of education		
Illiterate	12	40
Primary level	15	50
Secondary level	3	10
University	0	0

**Table 2:** Distribution of herbalists according to professional characteristics.

Variables	Number of respondents Proportion (%)	
Years of experience		
<6 years	2	6
7–12 years	6	20
13–20 years	15	50
>20 years	7	23
Source of acquisition of knowledge		
Ancestral experience	22	73
Radio	5	17
Books	2	7
TV	1	3

**Table 3:** Species of plants most requested by customers in the Fez-Boulemane region of Morocco.

Plant number	Botanical name	Families	Common name	Moroccan vernacular name	Frequency of citation
BTRL16E1	*Ammodaucus leucotrichus* Coss. and Dur.	Apiaceae	Ammodaucus	Kemmoun souffi	12
BTRL16E2	*Carum carvi* L.	Apiaceae	Caraway	Kerouiya	23
BTRL16E3	*Euphorbia resinifera* L.	Euphorbiaceae	Euphorbia	Daghmouss	9
BTRL16E4	*Herniaria fontanesii* L.	Caryophyllaceae	Smooth rupturewort	Herrast lehjar	20
BTRL16E5	*Lavandula coronopifolia* L.	Lamiaceae	Lavandula	Lekhzama	22
BTRL16E6	*Lawsonia inermis* L.	Lythraceae	Henna	L-henna	28
BTRL16E7	*Matricaria chamomilla* L.	Asteraceae	Camomile	Babounj	8
BTRL16E8	*Nigella sativa* L.	Ranunculaceae	Nigella	H'bat lbaraka, sanouj	16
BTRL16E9	*Origanum compactum* Benth L.	Lamiaceae	Oregano	Zaâtar, zoukni	21
BTRL16E10	*Pennisetum typhoides* L.	Poaceae	Pearl millet	Illan	13
BTRL16E11	*Rosmarinus officinalis* L.	Lamiaceae	Rosemary	Azel	26
BTRL16E12	*Ziziphus lotus* L.	Rhamnaceae	Ziziphus lotus	Sedra	10

**Table 4:** Distribution of pathologies treated with phytotherapy in Fez-Boulemane region, Morocco.

Pathology	Frequency of citation Proportion (%)	
Digestive disorders	42	25
Osteoarticular diseases	39	24
Urogenital diseases	21	12
Metabolic disorders	16	10
Skin disorders	10	6
Cardiovascular diseases	10	6
Parasitic diseases	8	5
Respiratory diseases	7	4
Food poisoning	6	4
Carcinogenic diseases	5	3
Liver disease	2	1

**Table 5:** Distribution of pathologies treated with phytotherapy in the Fez-Boulemane region of Morocco.

Ailment	Plants
Pathologies of digestive disorders	*Pennisetum typhoides* L., *Artemisia absinthium* L., *Ceratonia siliqua* L.,
	*Cinnamomum zeylanicum* L., *Citrullus colocynthis* L., *Citrus limonia* L.,
	*Coriandrum, Sativum* L., *Crocus sativus* L., *Fragaria vesca* L., *Mentha piperita* L.,
	*Myristica fragrans* Houtt., *Nigella sativa* L., *Ocimum basilicum* L., *Ormenis mixta* L.,
	*Papaver somniferum* L., *Peganum harmala* L., *Phoenix dactylifera* L.,
	*Pimpinella anisum* L., *Prunus amygdalus* L., *Punica granatum* L.,
	*Raphanus sativus* L., *Rosa centifolia* L., *Rosmarinus officinalis* L., *Ruta graveolens* L, *Salvadora persica* L., *Sesamum indicum* L., *Thymus vulgaris* L.,
	*Trigonella foenum-graecum* L., *Vitis vinifera* L., *Zea mays* L., *Zingiber officinale* L.
Metabolic disorders	*Artemisia absinthium* L., *Euphorbia resinifera* L., *Nerium oleander* L., *Artemisia*
	*herba-alba* Asso, *Brassica rapa* L., *Trigonella foenum-graecum,*
	*Glycyrrhiza glabra* L., *Armoracia rusticana* L., *Origanum majorana* L.,
	*Aristolochia baetica* L., *Salvia officinalis* L., *Cinnamomum verum* L., *Trigonella foenum-graecum*, *Linum usitatissimum* L., *Punica granatum* L., *Ficus carica* L.,
	*Eugenia caryophyllata* Thunb., *Olea europaea* L., *Nigella sativa* L.,
	*Rhamnus alaternus* L., *Prunus dulcis* L., *Ruta montana* L., *Zingiber officinale* L.
Respiratory diseases	*Atropa belladonna* L., *Brassica rapa* L., *Rosmarinus officinalis* L., *Viola odorata* L.,
	*Cinnamomum zeylanicum* L., *Eugenia caryophyllata* Thunb., *Mentha piperita* L.,
	*Marrubium vulgare* L., *Myristica fragrans* Houtt., *Ocimum basilicum* L., *Ormenis mixta* L., *Papaver rhoeas* L., *Papaver somniferum* L., *Petroselinum sativum* Hoffm.,
	*Phoenix dactylifera* L., *Pimpinella anisum* L., *Prunus amygdalus* L., *Thymus vulgaris* L., *Zingiber officinale* L.
Urogenital diseases	*Citrullus colocynthis* L., *Ocimum basilicum* L*., Ormenis mixta* L., *Papaver somniferum* L., *Peganum harmala* L., *Petroselinum sativum* Hoffm., *Pimpinella anisum* L., *Rosa centifolia* L., *Ruta graveolens* L., *Zingiber officinale* L., *Myristica fragrans* Houtt., *Atropa belladonna* L., *Olea europaea* L., *Petroselinum sativum* Hoffm., *Punica granatum* L., *Raphanus sativus* L., *Herniaria hirsuta* L., *Lavandula vera* L., *Peganum harmala* L.
Skin diseases	*Argania spinosa* L., *Citrus limonia* L., *Corylus avellana* L., *Lawsonia inermis* L.,
	*Peganum harmala* L., *Prunus amygdalus* Mill., *Raphanus sativus* L., *Rosa centifolia* L., *Ruta graveolens* L., *Sesamum indicum* L., *Atractylis gummifera* L., *Peganum harmala* L., *Lavandula vera* L., *Delphinium staphisagria* L., *Ziziphus lotus* L., *Thymelaea microphylla* L.
Cardiovascular diseases	*Allium sativum* L., *Argania spinosa* L., *Atropa belladonna* L., *Brassica rapa* L.,
	*Cinnamomum zeylanicum* L., *Corylus avellana* L., *Nigella sativa* L., *Olea europaea* L.,
	*Papaver rhoeas* L., *Punica granatum* L., *Rosmarinus officinalis* L.,
	*Sesamum indicum* L., *Thymus vulgaris* L., *Trigonella foenum-graecum* L.,
	*Viola odorata* L., *Vitis vinifera* L., *Prunus amygdalus* L.
Osteoarticular diseases	*Cinnamomum camphora* L., *Citrus limonia* L., *Myristica fragrans* Houtt.,
	*Papaver somniferum* L., *Rosmarinus officinalis* L., *Ruta graveolens* L.,
	*Thymus vulgaris* L., *Vitis vinifera* L., *Zea mays* L., *Zingiber officinale* L.,
	*Pennisetum typhoides* L., *Citrullus colocynthis* L., *Alpinia officinarum* Hance.

**Table 6:** Distribution of recommended toxic plants by herbalists in the region of Fez-Boulemane, Morocco.

Botanical name	Common name	Moroccan vernacular name	Frequency of citation	Proportion (%)
*Aristolochia longa* L.	Long aristolochi	Bereztem	14	11
*Atractylis gummifera* L.	Distaff thistle	Daad, chouk el aalk	20	15
*Atropa belladonna* L.	Belladonna	Belaydour	13	10
*Citrullus colocynthis* L.	Bitter apple	Hadja, taflzazte	11	8
*Delphinium staphisagria* L.	Stavesacre	Habat ras	9	7
*Myristica fragrans* Houtt.	Nutmeg	L'goza	4	3
*Nerium oleander* L.	Nerium	Deffla, Alili	15	11
*Nigella sativa* L.	Nigella	h'bat lbaraka, sanouj	7	5
*Papaver somniferum* L.	Opium poppy	Kharchakha	16	12
*Peganum harmala* L.	Wild rue	L'harmal	18	14
*Urginea maritima* L.	Squill	Âanssla	6	4

**Table 7:** Mixtures offered by herbalists in the region of Fez-Boulemane, Morocco.

Mixture	Common name	Botanical name	Mode of use
Mixture against cancer	Aristolochia baetica	*Aristolochia baetica* L.	All mixed with pure honey; it is advisable to take a small scoop every morning until healing
	*Fenugreek*	*Trigonella foenum-graecum* L.	
	*Euphorbia*	*Euphorbia resinifera L.*	
	White wormwood	*Artemisia herba-alba L.*	
	Ajuga iva	*Ajuga iva* L.	
	White horehound	*Marrubium vulgare* L.	
Mixture against digestive disorders	Black caraway	*Nigella sativa* L.	The plants are powdered and mixed with honey, a teaspoon on an empty stomach
	Anise	*Pimpinella anisum* L.	
	*Fennel*	*Foeniculum vulgare* L.	
Mixture against rheumatism	Nutmeg	*Myristica fragrans Houtt.*	All mixed with honey; it is advisable to take a spoonful of coffee 3 times a day before meals
	Ammodaucus	*Ammodaucus leucotrichus* Coss. and Dur.	
	Ginger	*Zingiber officinale* L.	
	Alpinia	*Alpinia officinarum.*L	
Mixture against hair loss	Wild rue	*Peganum harmala* L.	The two plants are mixed with olive oil and used as an antihair loss treatment. This mixture is preceded by onion juice application on the hair.
	Lavandula	*Lavandula coronopifolia* L.	
Mixture for the treatment of diabetes	Black caraway	*Nigella sativa* L.	Powdered *Nigella* mixes with dried white horehound and olive leaves and powdered madder roots
	White horehound	*Marrubium vulgare* L.	
	Olea europaea	*Olea europaea* L.	
	Rose madder	*Rubia tinctorum madder* L.	
Mixture in case of fever	Eucalyptus	*Eucalyptus globulus* L.	Fumigation
	White horehound	*Marrubium vulgare* L.	
	Ammi visnaga	*Ammi visnaga* L.	
	Dried cloves	*Syzygium aromaticum* L.	
	Turnip	*Brassica rapa* L.	
Mixture for ovarian stimulation	Sage	*Salvia officinalis* L.	Leaf decoction
	Oregano	*Origanum vulgare* L.	
	*Chamomile*	*Matricaria chamomilla* L.	
	*Fenugreek*	*Trigonella foenum-graecum* L.	
	Liquorice	*Glycyrrhiza glabra* L.	
	Alpinia	Alpinia*officinarum* L.	

## Data Availability

The data used to support the findings of this study are included within the article.
